# Inhibition of Vascular Endothelial Growth Factor Receptor 2 Exacerbates Loss of Lower Motor Neurons and Axons during Experimental Autoimmune Encephalomyelitis

**DOI:** 10.1371/journal.pone.0160158

**Published:** 2016-07-28

**Authors:** Milos Stanojlovic, Xiaosha Pang, Yifeng Lin, Sarrabeth Stone, Marija Cvetanovic, Wensheng Lin

**Affiliations:** 1 Department of Neuroscience, University of Minnesota, Minneapolis, Minnesota, United States of America; 2 Institute for Translational Neuroscience, University of Minnesota, Minneapolis, Minnesota, United States of America; University of Utah, UNITED STATES

## Abstract

Multiple sclerosis (MS) and its animal model experimental autoimmune encephalomyelitis (EAE) are inflammatory demyelinating and neurodegenerative diseases in the central nervous system (CNS). It is believed that MS and EAE are initiated by autoreactive T lymphocytes that recognize myelin antigens; however, the mechanisms responsible for neurodegeneration in these diseases remain elusive. Data indicate that vascular endothelial growth factor A (VEGF-A) plays a role in the development of MS and EAE. Interestingly, VEGF-A is regarded as a neurotrophic factor in the CNS that promotes neuron survival and neurogenesis in various neurodegenerative diseases by activating VEGF receptor 2 (VEGFR2). In this study, we sought to explore the role of the VEGF-A/VEGFR2 signaling in neurodegeneration in MS and EAE. We showed that the expression of VEGF-A was decreased in the spinal cord during EAE and that VEGFR2 was activated in lower motor neurons in the spinal cord of EAE mice. Interestingly, we found that treatment with SU5416, a selective VEGFR2 inhibitor, starting after the onset of EAE clinical symptoms exacerbated lower motor neuron loss and axon loss in the lumbar spinal cord of mice undergoing EAE, but did not alter Purkinje neuron loss in the cerebellum or upper motor neuron loss in the cerebral cortex. Moreover, SU5416 treatment had a minimal effect on EAE clinical symptoms as well as inflammation, demyelination, and oligodendrocyte loss in the lumbar spinal cord. These results imply the protective effects of the VEGF-A/VEGFR2 signaling on lower motor neurons and axons in the spinal cord in MS and EAE.

## Introduction

Multiple sclerosis (MS) and its animal model experimental autoimmune encephalomyelitis (EAE) are T-cell-mediated autoimmune diseases of the central nervous system (CNS), characterized by inflammatory demyelinated lesions in the white matter [1, 2]. The hallmarks of the demyelinated lesions in MS and EAE include inflammation, demyelination, oligodendrocyte loss, and axon degeneration. Interestingly, in the last few years, evidence has been emerging that neurodegeneration in the CNS gray matter is an early event and contributes to chronic disability in MS [3, 4]. Significant neuron loss has also been observed in the CNS gray matter of animals undergoing EAE, including the spinal cord, cerebral cortex, cerebellum, and hippocampus [5–8]. Although the current predominant view is that inflammation is ultimately responsible for axon degeneration and neuron loss in MS and EAE [9, 10], the molecular mechanisms responsible for neurodegeneration in these diseases remain largely unknown.

Vascular endothelial growth factor A (VEGF-A) is a potent endothelial cell growth factor, which stimulates blood vessel growth and regulates vascular permeability [11]. A number of studies have shown that VEGF-A increases angiogenesis and vascular permeability, and facilitates inflammation in various diseases [11, 12]. Data indicate the involvement of VEGF-A in regulating inflammation in MS and EAE, but these data are, at times, contradictory [13, 14]. Some reports showed that the level of VEGF-A is increased in MS and EAE lesions and the increased level of VEGF-A leads to enhanced inflammation in the CNS of EAE mice [14–16]. In contrast, other studies showed that the level of VEGF-A is decreased in the CNS of MS patients and EAE animals [17, 18]. Interestingly, intensive research in the last decade has shown that VEGF-A has direct effects on neurons and axons and functions as a neurotrophic factor that promotes neuron survival and neurogenesis in various neurodegenerative diseases, including amyotrophic lateral sclerosis, Alzheimer’s disease, Parkinson’s disease, spinocerebellar ataxia, and stroke [11, 19, 20]. VEGF-A exerts its functions through several receptors; one of these, VEGFR2, is believed to be involved in most of the neuron-specific functions [20, 21]. However, the role of the VEGF-A/VEGFR2 signaling in neurodegeneration in MS and EAE remains unexplored.

In this study, we sought to determine the effects of the VEGF-A/VEGFR2 signaling on neurodegeneration during EAE by treating mice with SU5416, a selective VEGFR2 inhibitor [22]. We found that SU5416 treatment starting after EAE onset exacerbated lower motor neuron loss and axon loss, but did not affect inflammation, demyelination, or oligodendrocyte loss in the lumbar spinal cord of EAE mice. Our finding implies a neuroprotective role of the VEGF-A/VEGFR2 signaling in lower motor neurons and axons in the spinal cord in MS and EAE.

## Materials and Methods

### EAE immunization and SU5416 treatment

To induce EAE, 7-weeks old C57BL/6J female mice were injected subcutaneously in the flank and at the tail base with 200 μg of myelin oligodendrocyte glycoprotein 35 to 55 (MOG 35–55) peptide emulsified in complete Freund’s adjuvant (BD Biosciences, San Jose, CA) supplemented with 600 μg of mycobacterium tuberculosis (strain H37Ra; BD Biosciences). Two intraperitoneal injections of 400 ng pertussis toxin (List Biological Laboratories, Denver, CO) were given 24 and 72 hours later. Clinical scores (0 indicates healthy; 1, flaccid tail; 2, ataxia and/or paresis of hind limbs; 3, paralysis of hind limbs and/or paresis of forelimbs; 4, tetra paralysis; and 5, moribund or death) were recorded daily as described in our previous papers [23–26]. SU5416 was purchased from Sigma-Aldrich (St. Louis, MO), and was dissolved in dimethyl sulfoxide (DMSO, Sigma-Aldrich). One group of mice received intraperitoneal injection of DMSO (30 μL) daily starting on the day after EAE disease onset to serve as controls. The other group of mice received intraperitoneal injection of 20 mg/kg SU5416 (30 μL) daily starting on the day after EAE disease onset. Mice were sacrificed at post-immunization day (PID) 21 through transcardial perfusion. Prior to transcardial perfusion, the animals were deeply anesthetized with intraperitoneal injections of 425 mg/kg Avertin (2,2,2-Tribromoethanol from Sigma-Aldrich). All mice were housed in the Research Animal Resources facilities of the University of Minnesota and received routine care, including feeding standard diets, providing fresh water, and changing cages and bedding. Animal health monitoring was performed on a daily basis by animal care staff, at least twice weekly by veterinary technicians and at least once weekly by a veterinarian. EAE mice were monitored starting at PID 1 twice daily. EAE mice that reached a score of 3.0 received supplemental nutrition, fluids and care on a twice daily basis. EAE mice that reached a score of 4.0 were sacrificed immediately. All animal procedures were conducted in complete compliance with the NIH Guide for the Care and Use of Laboratory Animals and were approved by the Institutional Animal Care and Use Committee (IACUC) of the University of Minnesota.

### VEGF-A ELISA analysis

Deeply anesthetized mice were perfused with ice-cold PBS. The spinal cords were harvested from mice, and homogenized using a motorized homogenizer, as previously described [23, 24]. After incubating on ice for 15 minutes, the extracts were cleared by centrifugation at 18,000g for 30 minutes, twice. The protein content of each extract was determined by DC Protein Assay (Bio-Rad Laboratories, Hercules, CA). The VEGF-A protein was measured using the Mouse VEGF-A ELISA kit (RayBiotech, Norcross GA), according to the manufacturer’s instructions.

### Histology and Immunohistochemistry

Anesthetized mice were perfused through the left cardiac ventricle with 4% paraformaldehyde in PBS. Brains were bisected in the sagittal plane. To isolate the lumbar spinal cord, the muscle and bone overlaying the dorsal side of the spinal cord from the sacral region (within the pelvis) to midway through thoracic region (middle of the rib cage) were carefully removed to expose the entire lumbar spinal cord, taking care not to severe the peripheral nerves. Lumber 1 –Lumber 5 peripheral nerves were identified based on the gross anatomy of the mouse as described in a previous paper [27]. The Lumber 3 nerve was identified and followed to the root at the spinal cord and the spinal cord was severed at the point where the Lumber 3 nerve entered the spinal cord. Both the upper (Lumber 1 –Lumber 3) and the lower (Lumber 3 –Lumber 5) regions of the lumbar spinal cord were carefully dissected from the vertebra.

One-half of brains and the spinal cord segments from the lumbar 3 to lumbar 5 were postfixed for at least 48 h in 4% paraformaldehyde in PBS, dehydrated through graded alcohols, and embedded in paraffin. Serial sections of 5 μm thickness were cut. Sections were routinely stained with hematoxylin and eosin (H&E). The other half of brains and the spinal cord segments from the lumbar 3 to lumbar 1 were postfixed for 1 h in 4% paraformaldehyde in PBS, cryopreserved in 30% sucrose for 48 h, embedded in OCT compound, and frozen on dry ice. Frozen sections were cut in a cryostat at 10 μm thickness. Immunohistochemistry (IHC) for VEGFR2 (1:100, Santa Cruz Biotechnology, Santa Cruz, CA), phosphorylated VEGFR2 (pVEGFR2,1:100, Santa Cruz Biotechnology), myelin basic protein (MBP, 1:1000, Sterberger monoclonals, Berkeley, CA), aspartoacylase (ASPA, 1:3000, kindly provided by Dr. M.A. Aryan Nomboodiri at Uniformed Services University of Health sciences, Bethesda, Maryland), NeuN (1:500, Millipore, Temecula, CA), phosphorylated neurofilament-H (SMI31, 1:1000, Sternberger Monoclonals), calbindin 2 (1:400, Sigma-Aldrich), CD3 (1:100, Santa Cruz Biotechnology), and CD11b (1:50; Millipore) were performed as previous described [25, 26, 28]. Signals were detected using fluorescein, Cy3, or enzyme-labeled secondary antibodies (Vector Laboratories, Burlingame, CA). Fluorescent-stained sections were mounted with Vectashield mounting medium with DAPI (Vector Laboratories) and visualized with a Zeiss Axioskop 2 fluorescence microscope (Carl Zeiss Microscopy, Thornwood, NY).

To quantify the cells and axons in the white matter, we counted immunopositive cells or axons within the anterior funiculus directly medial to the anterior median fissure in the lumbar spinal cord and confined to an area of 0.1 mm^2^, as described in our previous articles [24–26]. To quantify the lower motor neurons in the spinal cord segment from the lumbar 3 to lumbar 5, serial sections of 5 μm thickness were cut and every tenth section was immunostained with the NeuN antibody. The anterior horn of the spinal cord was selected for motor neuron counts. Only cells that had a visible nucleolus, the characteristic morphological features of an α-motor neuron, and a minimum diameter of 13.0 μm were counted using the NIH ImageJ software (http://rsbweb.nih.gov/ij/), as described in previous articles [29, 30].

To quantify the Purkinje neurons in the cerebellum, 5 μm thick sagittal brain sections were cut and every tenth section in the series spanning from Bregma lateral 0.12 mm to 0.36 mm were immunostained with the calbindin 2 antibody. Calbindin 2 positive cells were counted in the lobules I/II, III and IV of the anterior cerebellum as described in previous papers [31, 32]. To quantify the upper motor neurons in the primary motor cortex, 5 μm thick sagittal brain sections were cut and every tenth section in the series spanning from Bregma lateral 1.08 mm to 1.32 mm were immunostained with the NeuN antibody. NeuN positive cells were counted in the layer V of the primary motor cortex as described in a previous paper [33].

### Statistics

Data are expressed as mean ± standard deviation (SD). For quantitative analyses, multiple comparisons were statistically evaluated by the one-way ANOVA test followed by post hoc Bonferroni’s test using GraphPad Prism 5 (GraphPad Software). Comparison of two groups was statistically evaluated by *t*-test using GraphPad Prism 5. *P* < 0.05 was considered significant.

## Results

### VEGF-A/VEGFR2 signaling was activated in lower motor neurons in the spinal cord during EAE

**Fig 1:**
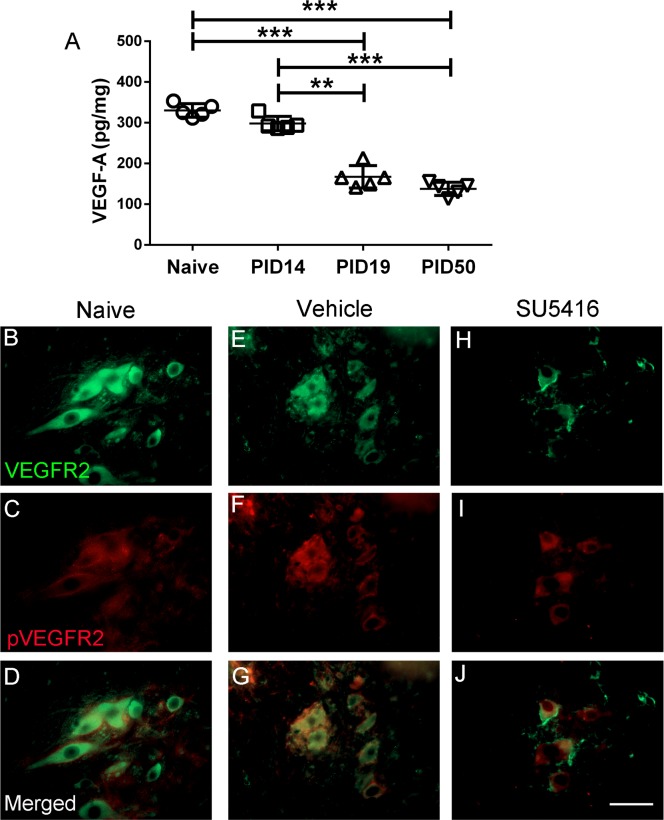
Activation of the VEGF-A/VEGFR2 signaling in lower motor neurons during EAE. **A**. VEGF-A ELISA showed that the protein level of VEGF-A was not altered in the spinal cord of EAE mice at PID14, but was significantly reduced at PID 19 and PID 50, as compared to naïve mice. N = 5 animals. **B—J**. VEGFR2 and pVEGFR2 double immunostaining revealed activation of VEGFR2 in lower motor neurons in the lumbar spinal cord of both naïve mice and EAE mice. Importantly, treatment with SU5416 noticeably reduced the levels of pVEGFR2 in the lower motor neurons of mice with EAE at PID 21. N = 5 animals. Error bars represent SD, **P < 0.01, ***P < 0.0001. Scale bar: B–J, 50 μm.

The data regarding the level of VEGF-A in the CNS of MS patients and EAE animals are contradictory. Some reports showed increased level of VEGF-A in MS and EAE [14–16]; however, other reports showed opposite results [17, 18]. We first determined the protein level of VEGF-A in the CNS during the course of EAE. Our previous studies showed that C57BL/6J mice immunized with MOG 35–55 peptide develop typical EAE disease course, the mice developed neurological signs of disease starting at approximately PID 12, reached the peak of disease around PID 19, and started recovering from EAE at approximately PID 22 [23–26]. ELISA analysis showed that VEGF-A level was not altered in the spinal cord of EAE mice at the onset of disease at PID14, but was significantly reduced at PID 19 (the acute phase of EAE) and PID 50 (the chronic phase of EAE), as compared to naïve mice (Fig 1A). These data suggest that VEGF-A level is decreased in the CNS during the course of EAE.

VEGF-A exerts direct actions on neurons by binding to VEGFR2, resulting in autophosphorylation of the receptor and subsequent activation of its downstream signaling pathways [11, 21]. It has been shown that lower motor neurons in the spinal cord express VEGFR2 and activation of the VEGF-A/VEGFR2 signaling is essential to lower motor neuron survival under physiological and pathological conditions [34, 35]. Loss of lower motor neurons has been observed in the lumbar spinal cord in MS patients and EAE animals [5, 36]. We examined the expression and activation of VEGFR2 in lower motor neurons during EAE. As expected, VEGFR2 and pVEGFR2 double immunostaining showed that lower motor neurons in the lumbar spinal cord of naïve mice express VEGFR2 and that the VEGFR2 was moderately activated (Fig 1B, 1C and 1D). However, the levels of both VEGFR2 and pVEGFR2 were not significantly altered in lower motor neurons of EAE mice as compared to naïve mice (Fig 1E, 1F and 1G). Thus, these data demonstrate activation of the VEGF-A/VEGFR2 signaling in lower motor neurons in the spinal cord during EAE.

### Impairment of VEGFR2 signaling after EAE onset exacerbated lower motor neuron loss and axon loss in lumbar spinal cord

SU5416 is a well-characterized, selective VEGFR2 inhibitor [22]. We assessed the effects of the VEGF-A/VEGFR2 signaling on EAE-induced neurodegeneration by treating mice with SU5416. Data suggest that activation of the VEGF-A/VEGFR2 signaling in endothelial cells increases the permeability of the blood brain barrier (BBB) and contributes to the infiltration of inflammatory cells in the CNS during EAE [14, 15]. Interestingly, a previous study showed that treatment with a high dose of SU5416 (50 mg/kg) during the acute phase of EAE suppresses inflammation in the CNS and attenuates EAE disease severity; however, the same treatment during the chronic phase of EAE does not affect inflammation or EAE disease severity [15]. The study raises the possibility that there is a window for SU5416 treatment during the course of EAE, in which the treatment does not affect inflammation, but has an impact on neurodegeneration.

**Fig 2:**
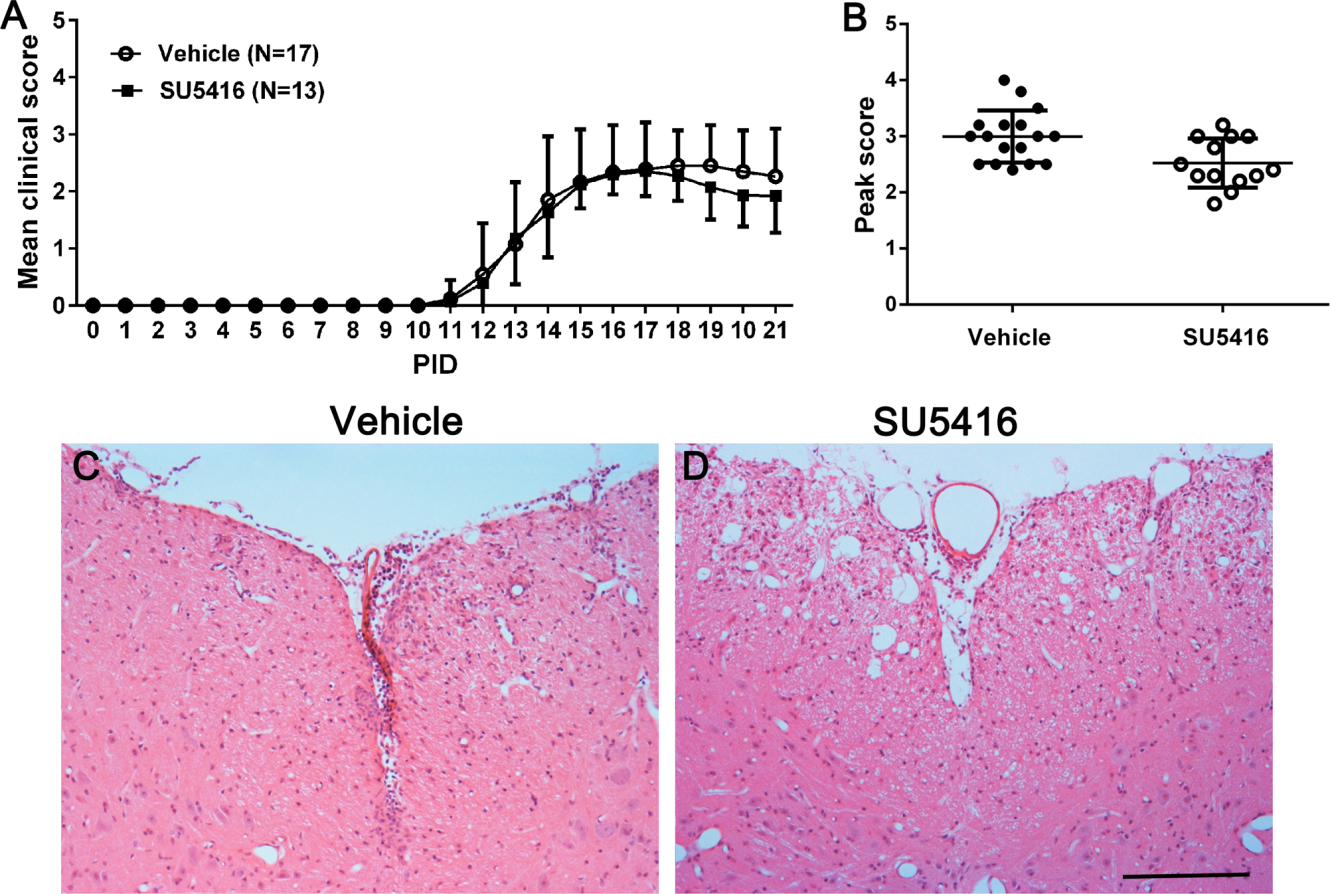
Treatment with the low dose of SU5416 after EAE onset did not alter the disease severity. **A.** Mean clinical score. **B.** Peak clinical score for individual mice. **C, D.** H&E staining revealed typical EAE pathology in the lumbar spinal of mice treated with vehicle and SU5416. N = 5 animals. Error bars represent SD. Scale bar: C, D, 200 μm.

A number of studies showed that BBB breakdown, inflammatory cell infiltration, and oligodendrocyte death occur in the CNS well before the onset of EAE clinical symptoms [25, 37]. Moreover, several reports showed that treatment with a low dose of SU5416 (10 mg/kg) is sufficient to impair the VEGF-A/VEGFR2 signaling in neurons and exacerbate neuron death in mouse models of brain injury [38, 39]. Thus, to minimize the impact of SU5416 treatment on inflammation in EAE mice, we used a low dose of SU5416 (20 mg/kg) and started the treatment on the day after EAE disease onset. We found that the disease severity displayed by SU5416-treated mice was comparable with vehicle-treated mice (Fig 2A and 2B). H&E staining showed comparable pathological changes in the lumbar spinal cord of these two groups of mice (Fig 2C and 2D). Unfortunately, we could not treat EAE mice with SU5416 beyond PID 21. Mice undergoing EAE did not tolerate daily intraperitoneal injection of DMSO (the vehicle) well. Our pilot study showed that all 3 SU5416-treated mice and all 3 vehicle-treated mice died after 10 days of daily intraperitoneal injections. All these mice died suddenly without obvious signs, besides typical EAE clinical symptoms. While occasional death of EAE mice was expected and was approved by the IACUC, we had not anticipated that all SU5416 or vehicle-treated mice died after 10 days of injections. The onset of EAE ranged from PID 11 to PID 14. Therefore, all EAE mice treated with SU5416 or vehicle were sacrificed at PID 21. None of SU5416 or vehicle-treated mice died unexpectedly by PID 21, and one of vehicle-treated mice reached a clinical score of 4.0 were sacrificed immediately (Fig 2A and 2B). The CNS tissues were preserved for either histological studies or biochemical studies.

**Fig 3:**
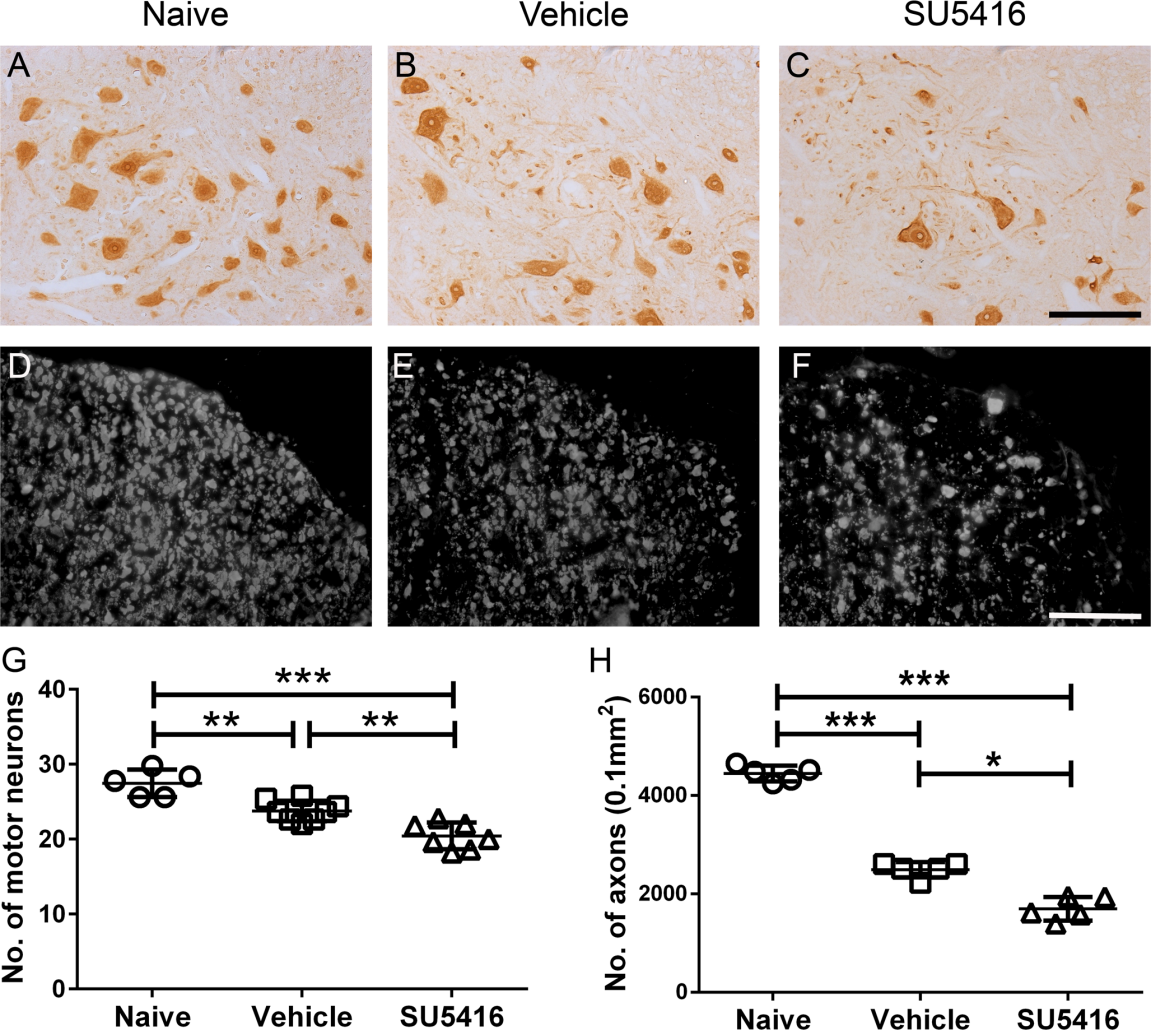
Treatment with the low dose of SU5416 after EAE onset exacerbated lower motor neuron loss and axon loss in the lumbar spinal cord. **A, B, C, G.** NeuN IHC showed that the number of lower motor neurons was significantly reduced in the lumbar spinal cord of vehicle-treated EAE mice at PID 21 as compared to naïve mice, and that SU5416 treatment further reduced the motor neuron numbers in SU5416-treated EAE mice. N = 5–7 animals. **D, E, F, H.** SMI31 IHC showed that the number of axons was significantly reduced in the lumbar spinal cord of vehicle-treated EAE mice at PID 21 as compared to naïve mice, and that SU5416 treatment further reduced the axon numbers in SU5416-treated EAE mice. N = 5 animals. Error bars represent SD, *P < 0.05, **P < 0.001,***P < 0.0001. Scale bar: A–C, 100 μm; D–F, 25 μm.

Next, we examined the effects of SU5416 treatment on EAE-induced neuron loss and axon loss in the CNS. In agreement with previous studies [38, 39], we found that treatment with the low dose of SU5416 (20mg/kg) noticeably reduced the immunoreactivity of pVEGFR2 in lower motor neurons in the lumbar spinal cord of mice undergoing EAE (Fig 1H, 1I and 1J). Consistent with previous studies [5, 36], NeuN IHC showed a significant reduction of lower motor neuron numbers in the lumbar spinal of vehicle-treated mice at PID 21 as compared to naïve mice (Fig 3A, 3B and 3G). Importantly, the number of lower motor neurons was further reduced in the lumbar spinal cord of SU5416-treated mice (Fig 3B, 3C and 3G). Moreover, phosphorylated neurofilament-H (SMI-31) IHC revealed a significant reduction of axon numbers in the white mater in the lumbar spinal cord of vehicle-treated mice at PID 21 as compared to naïve mice (Fig 3D, 3E and 3H). Interestingly, the number of axons was further reduced in the lumbar spinal cord of SU5416-treated mice (Fig 3E, 3F and 3H). Taken together, these results suggest that SU5416 treatment attenuates activation of VEGFR2 signaling in lower motor neurons, and exacerbates loss of lower motor neurons and axons in the spinal cord during EAE.

**Fig 4:**
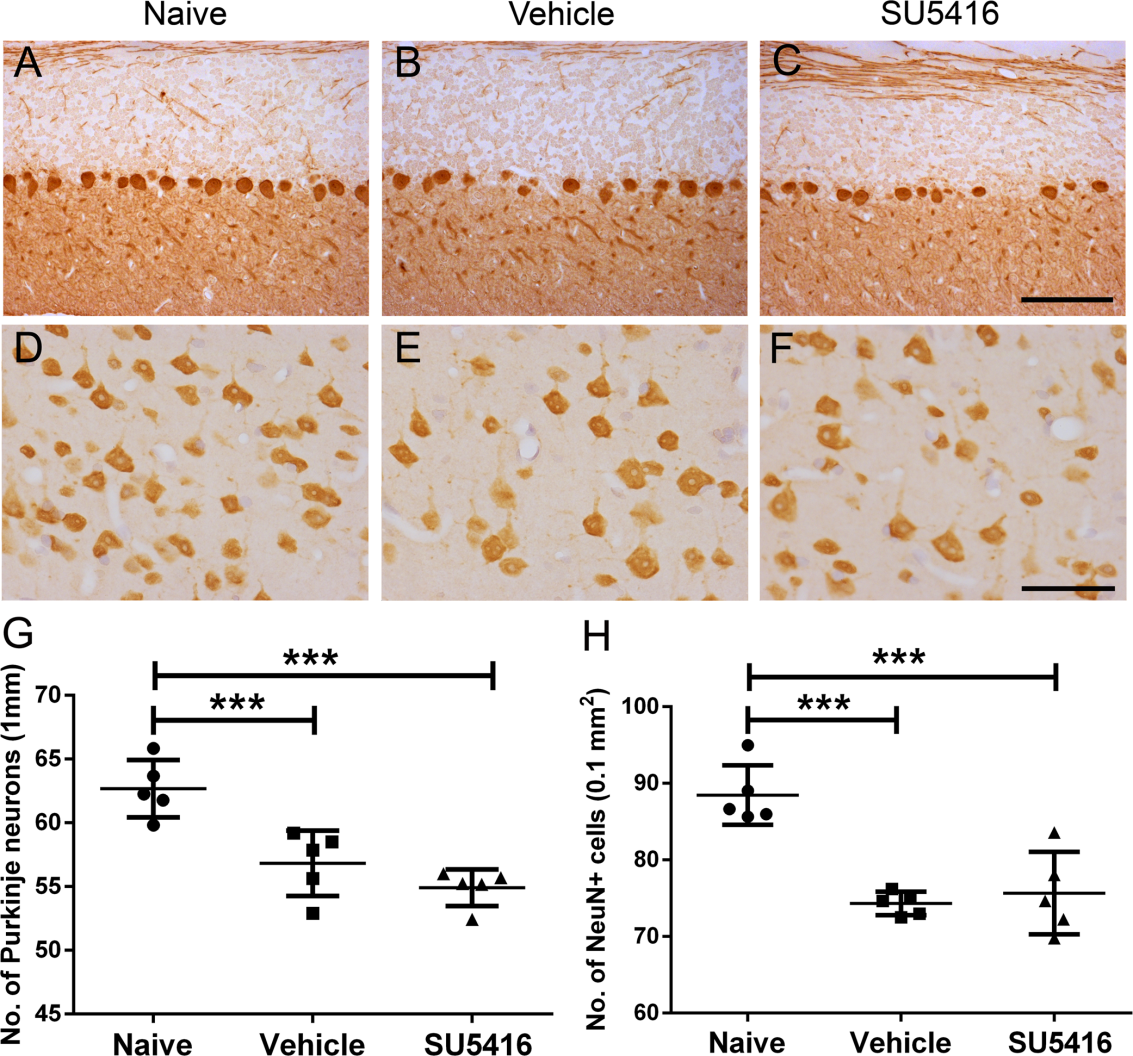
Treatment with the low dose of SU5416 after EAE onset did not affect Purkinje neuron loss or upper motor neuron loss. **A, B, C, G.** Calbindin 2 IHC showed the number of Purkinje neurons was significantly reduced in the cerebellum of vehicle-treated EAE mice at PID 21 as compared to naïve mice, and that SU5416 treatment did not significantly change the number of Purkinje neurons in the cerebellum of EAE mice. N = 5 animals. **D, E, F, H.** NeuN IHC showed the number of neurons in the layer V of the primary motor cortex was significantly reduced in vehicle-treated EAE mice at PID 21 as compared to naïve mice, and that SU5416 treatment did not change the number of neurons in the layer V of the primary motor cortex of EAE mice. N = 5 animals. ***P < 0.0001. Scale bar: A–C, 100 μm; D–F, 50 μm.

In contrast, although we found the significant reduction of Purkinje neuron numbers in the cerebellum of EAE mice as compared to naïve mice (Fig 4A, 4B and 4G), SU5416 treatment did not significantly alter Purkinje neuron loss during EAE (Fig 4B, 4C and 4G). Similarly, we found that SU5416 treatment did not significantly affect EAE-induced upper motor neuron loss in the layer V of the primary motor cortex (Fig 4D, 4E, 4F and 4H). Taken together, these data likely reflects that the VEGF-A/VEGFR2 signaling is critical for the survival of lower motor neurons and axons in the spinal cord during EAE, but has no major effect on the viability of Purkinje neurons or upper motor neurons.

### Treatment with SU5416 after EAE onset did not significantly affect inflammation or demyelination in the lumbar spinal cord

**Fig 5:**
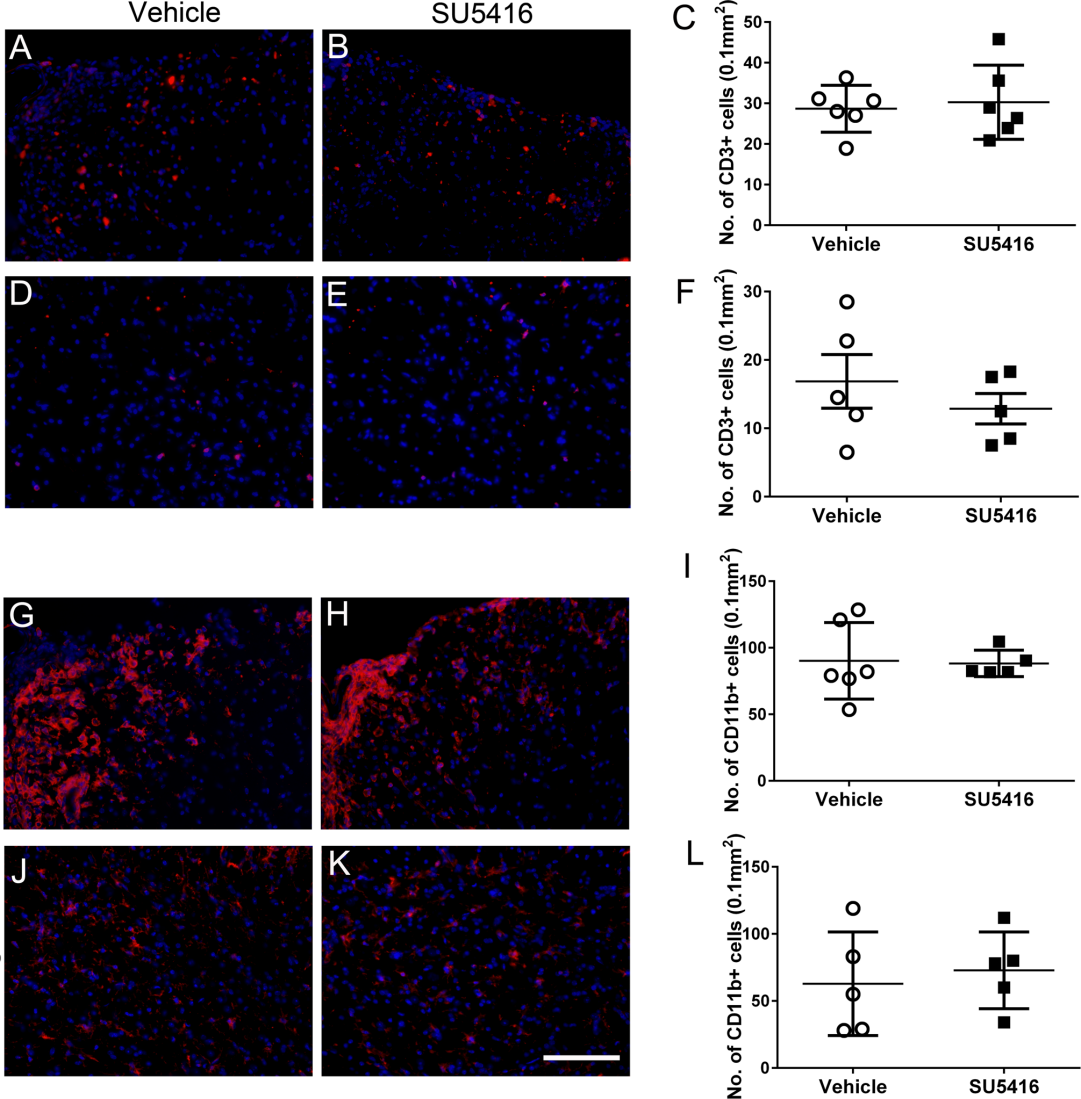
Treatment with the low dose of SU5416 after EAE onset did not change the infiltration of inflammatory cells in the lumbar spinal cord during EAE. **A, B, C.** CD3 immunostaining showed comparable numbers of CD3 positive T cells in the white matter in the lumbar spinal cord of vehicle-treated mice and SU5416-treated mice at PID 21. N = 5 animals. **D, E, F.** CD3 immunostaining showed comparable numbers of CD3 positive T cells in the anterior horn in the lumbar spinal cord of vehicle-treated mice and SU5416-treated mice at PID 21. N = 5 animals. **G, H, I.** CD11b immunostaining showed comparable numbers of CD11b positive microglia/macrophages in the white matter in the lumbar spinal cord of vehicle-treated mice and SU5416-treated mice at PID 21. N = 5 animals. **J, K, L.** CD11b immunostaining showed comparable numbers of CD11b positive microglia/macrophages in the anterior horn in the lumbar spinal cord of vehicle-treated mice and SU5416-treated mice at PID 21. N = 5 animals. Error bars represent SD. Scale bar: A, B, D, E, G, H, J, K, 100 μm.

As described above, a previous report showed that the effects of SU5416 treatment on inflammation during the course of EAE is determined by the timing of treatment [15]. We examined whether treatment with the low dose of SU5416 (20 mg/kg) starting after EAE disease onset influenced infiltration of inflammatory cells in the CNS. CD3 immunostaining showed that SU5416 treatment did not significantly alter the number of infiltrated T cells in either the white matter or gray matter in the lumbar spinal cord at PID 21 (Fig 5A, 5B, 5C, 5D, 5E and 5F). Moreover, CD11b immunostaining showed that SU5416 treatment did not significantly change the number of microglia/macrophages in either the white matter or gray matter in the lumbar spinal cord at PID 21 (Fig 5G, 5H, 5I, 5J, 5K and 5L). Collectively, these data suggest that the low dose of SU5416 treatment after EAE onset has no significant impact on inflammation in the spinal cord.

**Fig 6:**
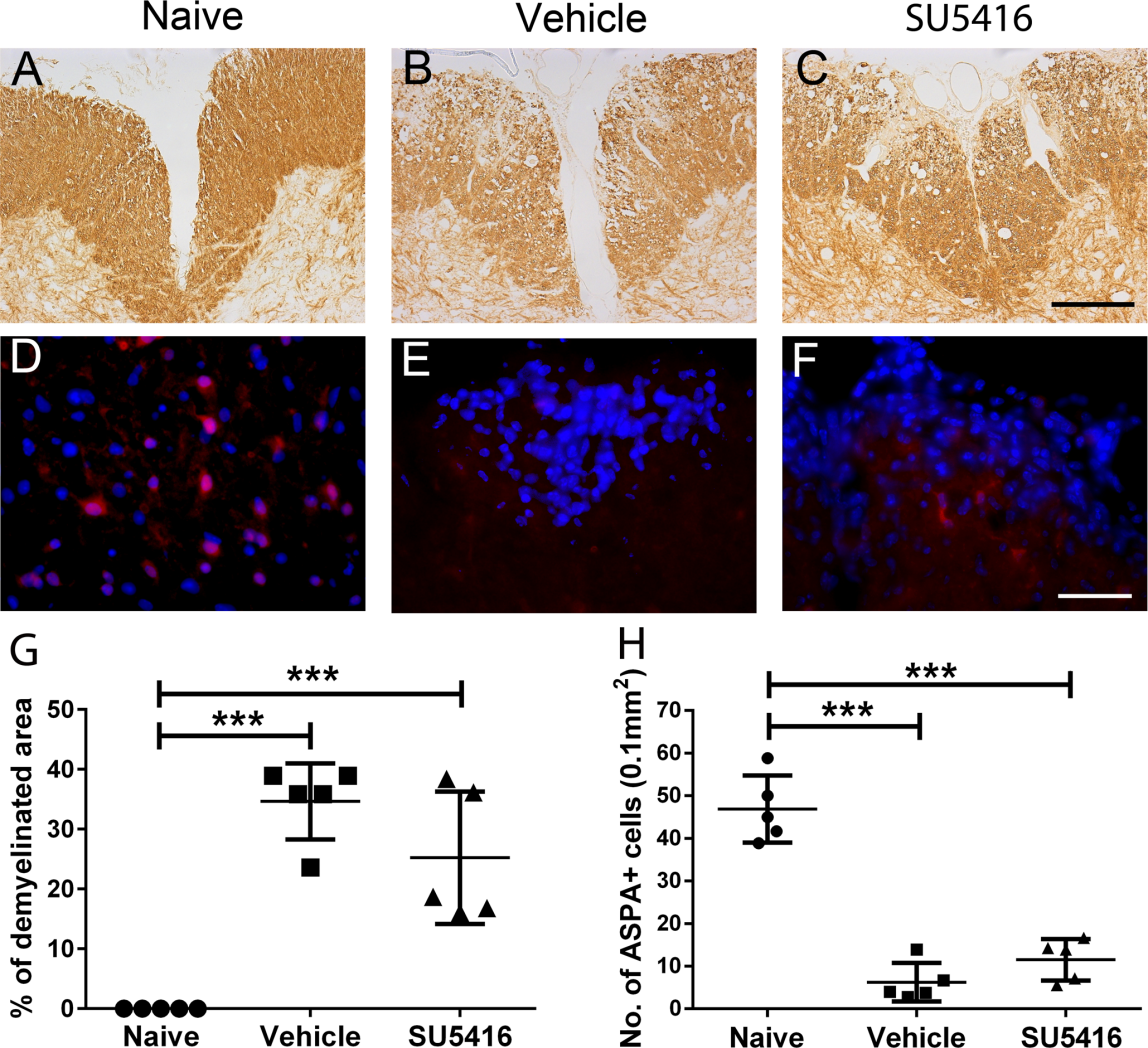
Treatment with the low dose of SU5416 after EAE onset did not affect demyelination or oligodendrocyte loss in the lumbar spinal cord during EAE. **A, B, C, G.** MBP IHC showed that treatment with the low dose of SU5416 after EAE onset did not significantly change the degree of demyelination in the lumbar spinal cord at PID 21. N = 5 animals. **D, E, F, H.** ASPA immunostaining showed that treatment with the low dose of SU5416 after EAE onset did not significantly change the degree of reduction of oligodendrocyte numbers in the lumbar spinal cord at PID 21. N = 5 animals. Error bars represent SD, ***P < 0.0001. Scale bar: A–C, 200 μm; D–F, 50 μm.

Furthermore, we determined the effects of SU5416 treatment on demyelination and oligodendrocyte loss in the lumbar spinal cord of EAE mice. MBP IHC showed comparable myelin damage in the lumbar spinal cord of vehicle-treated mice and SU5416-treated mice at PID 21 (Fig 6A, 6B and 6C). We quantified the percentage of the white matter area that was demyelinated in the lumbar spinal cord by normalizing the demyelinated white matter area against the total white matter area. We found that SU5416 treatment did not significantly alter the percentage of demyelinated area in the lumbar spinal cord at PID 21 (Fig 6G). Similarly, immunostaining for ASPA, a maker for oligodendrocytes [25, 40, 41], showed that oligodendrocyte numbers were significantly reduced in the lumbar spinal cord of vehicle-treated mice at PID 21 as compared to naïve mice, and that SU5416 treatment did not significantly change the number of oligodendrocytes in the lumbar spinal cord of EAE mice (Fig 6D, 6E, 6F and 6H). Thus, these data suggest that inhibition of VEGFR2 signaling after EAE onset did not significantly influence EAE-induced demyelination or oligodendrocyte loss in the spinal cord.

## Discussion

VEGF-A was originally identified as an endothelial cell specific growth factor. Interestingly, recent studies showed that VEGF-A plays an important role in the CNS under normal and disease conditions [11, 20]. VEGF-A exerts direct actions on various CNS cell types, including neural progenitor cells, neurons, astrocytes, oligodendrocyte progenitor cells (OPCs), and microglia, by activating its receptor, VEGFR2 [21]. It has been shown that activation of the VEGF-A/VEGFR2 signaling promotes neuron survival and neurogenesis in various neurodegenerative diseases [11, 20]. MS and EAE are chronic inflammatory demyelinating and neurodegenerative diseases of the CNS [3, 4]. Although there is evidence that the VEGF-A/VEGFR2 signaling regulates inflammation during EAE [13, 14], the role of this pathway in neurodegeneration in this disease has not been explored. Using a selective VEGFR2 inhibitor SU5416, in this study, we showed that treatment with the low dose of SU5416 (20 mg/kg) starting after EAE onset significantly exacerbated lower motor neuron loss and axon loss, but did not significantly affect inflammation, demyelination, or oligodendrocyte loss in the lumbar spinal cord of EAE mice. These results provide the first evidence that activation of the VEGF-A/VEGFR2 signaling protects neurons and axons against inflammation in MS and EAE.

Data indicate that the VEGF-A/VEGFR2 signaling is essential for lower motor neuron survival in the spinal cord under physiological conditions [34, 35]. This pathway also promotes lower motor neuron survival under pathological conditions [35, 42]. In agreement with these studies, we showed herein that VEGFR2 was activated in lower motor neurons in the lumbar spinal cord of mice undergoing EAE, and that treatment with the low dose of SU5416 impaired VEGFR2 activation and resulted in exacerbation of EAE-induced lower motor neuron loss. Moreover, we found that SU5416 treatment exacerbated EAE-induced axon loss in the spinal cord. In contrast, we showed that SU5416 treatment did not affect Purkinje neuron loss in the cerebellum or upper motor neuron loss in the cerebral cortex during EAE. Collectively, these results raise the possibility that the effects of the VEGF-A/VEGFR2 signaling on neuron viability during EAE are neuron-type dependent. The VEGF-A/VEGFR2 signaling modulates the viability of certain neuron types during EAE, including lower motor neurons in the spinal cord; however, other neuron-types, such as Purkinje neurons and upper motor neurons, are not sensitive to this signaling. On the other hand, our intent is to minimize the impact of SU5416 treatment on inflammation during EAE mice by using the low dose of SU5416. Therefore, the alternative possibility is that the low dose of SU5416 treatment is sufficient to block the VEGFR2 signaling in lower motor neurons in the lumbar spinal cord, but is not sufficient to impair the VEGFR2 signaling in other types of neurons. Unfortunately, the results presented in this study do not allow us to dissect the precise role of the VEGFR2 signaling on different neuron types in the CNS during EAE. Clearly, the role of VEGFR2 signaling on neurons in MS and EAE warrants further investigation. A neuron-type specific conditional mouse model that allows for inactivation of VEGFR2 selectively in specific type of neurons would be an ideal model to address this important open question.

Although data indicate that activation of the VEGF-A/VEGFR2 signaling in endothelial cells leads to infiltration of inflammatory cells in the CNS by increasing the permeability of the BBB [14, 43], several studies demonstrated that the effects of this signaling on inflammatory cell infiltration during the course of CNS diseases are determined by the timing of activation [15, 44, 45]. A study showed that treatment with a high dose of SU5416 (50 mg/kg) during the acute phase of EAE suppresses inflammation in the CNS; however, the same treatment during the chronic phase of EAE has no effect on inflammation [15]. The goal of this study is to dissect the effects of the VEGF-A/VEGFR2 signaling on neurodegeneration during EAE. It is believed that inflammation contributes to neurodegeneration in MS and EAE [9, 10]. Therefore, we tried our best to minimize the effects of SU5416 treatment on inflammation during EAE. We tested various doses of SU5416 and various timings for the treatment. After considerable effort, we found that treatment with the low dose of SU5416 (20mg/kg) starting after EAE onset did not significantly alter inflammation, but noticeably impaired VEGFR2 signaling in the lower motor neurons and significantly exacerbated EAE-induced lower motor neuron loss and axon loss in the spinal cord. Since it is known that BBB breakdown and inflammatory cell infiltration occur in the CNS well before the onset of EAE clinical symptoms [25, 37], these data likely reflects that SU5416 treatment starting after EAE onset is too late to have an impact on the permeability of the BBB and subsequent inflammatory cell infiltration. Moreover, several studies have reported that lower motor neurons are more sensitive to the alternation of the VEGF-A/VEGFR2 signaling than endothelial cells [34, 44, 45]. Thus, an alternative, but not mutually exclusive, possibility is that impairment of the VEGF-A/VEGFR2 signaling induced by the low dose of SU5416 (20 mg/kg) treatment is sufficient to increase the sensitivity of lower motor neurons to inflammation during EAE, but is not sufficient to alter the function of endothelial cells and influence inflammation in the CNS.

While previous studies showed that OPCs express VEGFR2 and that VEGF-A promotes OPC migration by activating VEGFR2 [46, 47], there is no evidence that the VEGF-A/VEGFR2 signaling influences differentiated oligodendroglia under normal or disease conditions. It is generally believed that inflammation is responsible for oligodendrocyte death and myelin damage during EAE [1, 2, 4]. We showed herein that treatment with the low dose of SU5416 (20mg/kg) starting after EAE onset had no major effect on inflammation. Not surprisingly, we found that SU5416 treatment did not alter oligodendrocyte loss or demyelination during EAE. Moreover, we found that the treatment did not significantly affect the severity of EAE clinical symptoms. There is evidence suggesting that inflammation and demyelination contributes significantly to the clinical symptoms of EAE [48]. Since SU5416 treatment did not alter the degree of inflammation, demyelination, or oligodendrocyte loss in the CNS of mice undergoing EAE, it is likely that the lack of effect of SU5416 treatment on EAE clinical symptoms is due to its minimal actions in inflammation and demyelination in the CNS of EAE mice. On the other hand, it is well documented that the CNS has an ability to compensate for greater than 50% loss of specific types of neurons, without displaying clinical symptoms (compensated state) [49]. Neurodegeneration in the CNS at the acute phase of EAE is modest (~ 20% loss of neurons), far away from the threshold of the decompensated state of the CNS. Additionally, we showed that SU5416 treatment significantly but moderately exacerbated neurodegeneration in the lumbar spinal cord of EAE mice. It is unlikely that SU5416 treatment drives neurodegeneration in the CNS of EAE mice to the threshold of the decompensated state. Not surprisingly, moderate exacerbation of neuron loss and axon loss induced by SU5416 did not significantly contribute to the EAE clinical symptoms.

It has been shown that all major CNS cell types express VEGF-A, including neurons, astrocytes, oligodendrocytes, microglia, and endothelial cells [50]. The previous data regarding VEGF-A level in the CNS of EAE animals are contradictory. Some reports showed the increased level of VEGF-A in the CNS during EAE [14–16]; however, other reports showed opposite results [17, 18]. There are a number of different EAE models. Each of these models displays very different disease course and CNS pathology. These reports use different EAE models as well as different methods to measure the level of VEGF-A. The contradictory data likely result from different model systems and/or different measurement methods. Using the well-characterized MOG35-55 EAE model as well as the highly sensitive and reproducible ELISA assay, we showed here that the level of VEGF-A in the CNS was not altered at the onset of disease, but was decreased at the peak of disease and the chronic phase of disease. Our data demonstrate the level of VEGF-A is decreased in the CNS in the MOG35-55 EAE model at the both acute and chronic phases of disease. Our results also suggest that the decreased level of VEGF-A may contribute to loss of lower motor neurons and axons in the spinal cord during EAE.

In summary, the results presented herein suggest the protective effects of the VEGF-A/VEGFR2 signaling on lower motor neurons and axons in the spinal cord during EAE. Neurodegeneration is considered to be the primary cause of chronic disability in MS. However, the molecular mechanisms responsible for neurodegeneration in MS and EAE remain largely unknown. There is no available therapy for MS that attenuates neuron loss and/or axon loss [51, 52]. This study implicates that therapeutic strategies that activate the VEGFR2 signaling in neurons may be beneficial to MS patients.
